# Pharmacokinetics of Repeated Oral Dosing with Coenzyme Q10 in Cavalier King Charles Spaniels with Myxomatous Mitral Valve Disease

**DOI:** 10.3390/antiox9090827

**Published:** 2020-09-04

**Authors:** Liselotte B. Christiansen, Malene K. Morsing, Maria Josefine Reimann, Torben Martinussen, Zita Birlie, Anne Marie V. Schou-Pedersen, Jens Lykkesfeldt, Lisbeth H. Olsen

**Affiliations:** 1 Section for Experimental Animal Models, Department of Veterinary and Animal Sciences, University of Copenhagen, Ridebanevej 9, 1870 Frederiksberg C, Denmark; makm@sund.ku.dk (M.K.M.); mreimann@sund.ku.dk (M.J.R.); zitaberiksen@gmail.com (Z.B.); am.schoupedersen@sund.ku.dk (A.M.V.S.-P.); jopl@sund.ku.dk (J.L.); lisbeth.hoier@sund.ku.dk (L.H.O.); 2 Section for Biostatistics, Department of Public Health, Faculty of Health and Medical Sciences, University of Copenhagen, Øster Farimagsgade 5B, 1014 Copenhagen K, Denmark; tma@sund.ku.dk

**Keywords:** dog, degenerative mitral valve disease, congestive heart failure, antioxidant, ubiquinone

## Abstract

Coenzyme Q10 (Q10) is a mitochondrial cofactor and an antioxidant with the potential to combat oxidative stress in heart failure. This study aims to determine the pharmacokinetics of repeated oral dosing of Q10 in Cavalier King Charles Spaniels (CKCS) with spontaneous myxomatous mitral valve disease (MMVD) and to evaluate echocardiographic parameters, circulating cardiac biomarkers, and quality of life (QoL) after treatment. The study is a randomized, placebo-controlled, single-blinded crossover study. Nineteen CKCS with MMVD were randomized to receive 100 mg Q10 (ubiquinone) bi-daily for three weeks, then placebo (or in reverse order). Clinical examination, blood sampling, echocardiography, and QoL assessment were performed before and after each treatment phase. Q10 plasma concentrations were determined in plasma using a validated high-performance liquid chromatography method using electrochemical detection (HPLC-ECD). Eighteen CKCS were included in the analyses. Total plasma concentration of Q10 increased significantly (*p* < 0.0001) from baseline (median, 0.92 µg/mL; interquartile range (IQR), 0.70–1.26) to after treatment (median, 3.51 µg/mL; IQR, 2.30–6.88). Thirteen dogs reached the threshold of a total plasma Q10 concentration of ≥2.0 µg/mL. The average half-life (T_1/2_) of Q10 was 2.95 days (IQR, 1.75–4.02). No significant differences were observed in clinical MMVD severity, and the owner perceived QoL between Q10 and placebo treatment. The solubilized Q10 formulation was well-tolerated in the dogs. Individual variation in plasma concentrations was observed following oral treatment. A long-term placebo-controlled trial is warranted in dogs with MMVD to determine long-term efficacy on the clinical severity of MMVD.

## 1. Introduction

Myxomatous mitral valve disease (MMVD) is the most common heart disease in dogs [1] and an important cause for the development of congestive heart failure (CHF). The Cavalier King Charles Spaniel (CKCS) is a breed especially prone to develop MMVD at an early age [2,3]. The median survival time from onset of CHF in dogs with MMVD is less than 12 months when using the currently recommended medical therapy [4]. To increase the life expectancy and quality of life (QoL) in dogs with severe MMVD, novel treatment strategies are needed.

Coenzyme Q10 (Q10) is a vitamin-like, lipophilic endogenous substance with high abundance in the mitochondrial membranes of all organs [5]. Q10 exists as a redox couple consisting of reduced (ubiquinol) and oxidized (ubiquinone) fractions between which an in vivo equilibrium exists. Q10 serves two primary functions: (i) Being an energy transferring molecule in mitochondrial oxidative phosphorylation, ubiquinone assists in the production of adenosine triphosphate (ATP), which is used as energetic fuel in cellular processes, including muscle contraction and relaxation [6], and (ii) ubiquinol is a potent antioxidant, protecting cells from oxidative damage from free radicals [7]. In the circulating blood, the vast majority of Q10 exists as ubiquinol [8].

Mitochondrial dysfunction and oxidative stress are known to play a role in the progression of heart failure [9,10,11]. The rationale for using Q10 as a dietary supplement in patients with heart failure is to support mitochondrial function and combat excessive amounts of reactive oxygen species. By raising the endogenous Q10 concentration above a certain level using oral supplementation, it has been shown that myocardial Q10 concentrations increase accordingly [12]. The availability of orally delivered Q10 to the heart muscle is primarily by micellar absorption in the small intestine, transport through the enterocytes by yet unknown transport mechanisms, and attachment to chylomicrons, which are taken up by the lymphatic system and passaged to the systemic circulation [13]. 

A minimum concentration of total circulating Q10 concentration of 2.0 μg/mL was proposed as a relevant target concentration in human heart failure patients [14]. This is above normal endogenous circulating concentrations reported in both healthy adult humans (means of serum Q10 concentration range: 0.6–1.0 μg/mL) [15] and in dogs (0.4–1.5 μg/mL) [16,17]. In a recent clinical trial done in human heart failure patients (Q-SYMBIO), the 2.0 μg/mL concentration of total circulating Q10 was used as a target with the administration of 300 mg ubiquinone daily and showed a positive effect of Q10 on clinical endpoints [18]. The mechanisms underlying the demonstrated clinical efficacy in patients with congestive heart failure are currently not known. 

To the best of our knowledge, there are no previous studies evaluating pharmacokinetic properties and clinical efficacy of Q10 in dogs with naturally occurring heart disease or heart failure. The aim of this study, therefore, was twofold: (i) To analyze the basic pharmacokinetic properties of repeated oral dosing of Q10 formulated as ubiquinone solubilized in vegetable oil and (ii) to investigate if short-term repeated oral dosing with Q10 has a positive effect on clinical parameters of MMVD and QoL in CKCS. 

We hypothesized as the primary endpoint that a total Q10 plasma concentration of ≥2.0 μg/mL would be reached with oral Q10 supplement (100 mg twice daily) in CKCS. Secondly, we hypothesized that treatment with Q10 would enhance the QoL in dogs with severe heart disease and improve clinical endpoints related to clinical MMVD severity, including echocardiographic measures and circulating cardiac biomarkers. 

## 2. Materials and Methods 

### 2.1. Animals

The study was a randomized, single-blinded crossover clinical trial comparing ubiquinone (Q10) treatment with a placebo. The study was conducted at the Department of Veterinary and Animal Sciences, University of Copenhagen. The study protocol was approved by the Experimental Animals Inspectorate (License number: 2016-15-0201-01074) and by the Danish Medicines Agency, and written informed consent was obtained from owners of dogs enrolled in the study. 

Inclusion criteria were: Pedigree-bred CKCS above four years of age with a diagnosis of MMVD classified as American College of Internal Medicine (ACVIM) stage B2 or C according to previously described criteria [19]: Left ventricular internal diameter at end-diastole normalized to bodyweight (LVIDDN) ≥1.7, left atrial to aortic root ratio (LA/Ao) ≥1.6, vertebral heart sum (VHS) >10.5 and presence of a characteristic systolic heart murmur with minimum 3/6 in intensity. 

Dogs were excluded if they had echocardiographic signs of other cardiac diseases than MMVD, any signs of significant systemic disease on blood work and clinical examination, or respiratory disease, was revealed by thoracic X-rays. Pregnancy and lactation were also exclusion criteria, as was the use of corticosteroids and nutritional supplements containing antioxidants for at least 30 days. Treatment with other types of medication, including cardiac treatment, did not exclude the dogs—but treatment dose and frequency had to be unchanged for at least 30 days prior to study start and throughout the entire study period. 

### 2.2. Experimental Design

**Figure 1:**
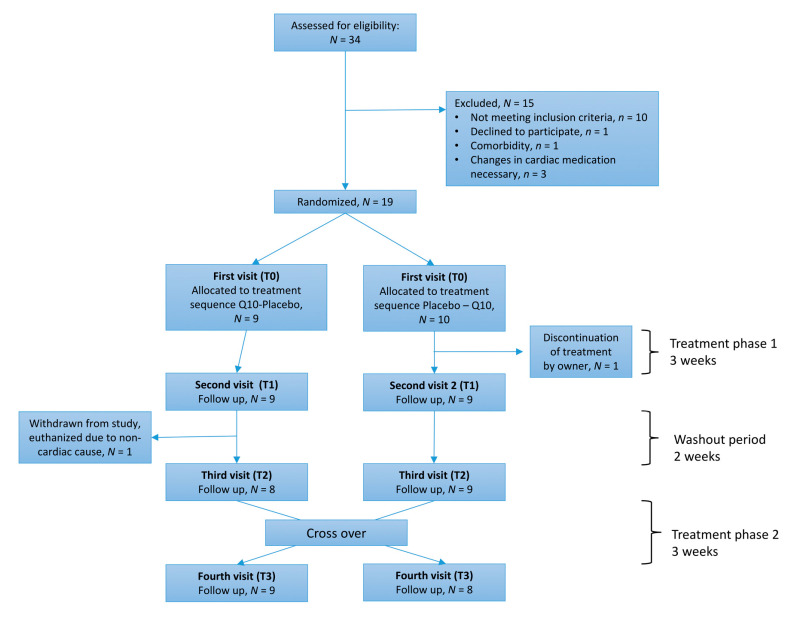
Flowchart of randomized placebo-controlled crossover study design. A modified CONSORT template [21] was used. Nineteen Cavalier King Charles Spaniels were randomized to one of two treatment arms: Q10 first, placebo second, or placebo first, Q10 second. Seventeen dogs completed the entire study.

Dogs were randomized in blocks of four dogs into one of two groups (Q10 in the first phase with crossover to placebo or placebo first with crossover to Q10) according to a randomization plan generated using the website www.randomization.com [20]. The first phase was followed by a 2-week washout period. The order of treatments was blinded to the owner of the dog. Dogs presented in the clinic at four visits: Before (T0) and after (T1) the first treatment phase and before (T2) and after (T3) the second treatment phase (Figure 1).

Prior to the examinations, dogs were food fasted for 6–18 h. Initially, at each visit, the history of the dog was recorded, and bodyweight (BW) was obtained. A questionnaire evaluating the owner’s perception of their dogs’ QoL during the past seven days was completed, either on arrival to the clinic or at home, prior to the visit, for ensuring that the same family member answered the questionnaire each time. One owner of each dog was asked to grade the following items: Exercise intolerance, demeanor, appetite, nocturnal dyspnea, cough, respiratory effort, and syncope, as previously described with modification [19]. An overall score ranging from 7 to 28 was obtained with the lowest score indicating the best QoL. 

Using the same standardized procedures for each of the visits (T0–T3), the dogs then underwent a clinical examination, blood sampling, echocardiography, and blood pressure measurement using high-definition oscillometry (High Definition Oscillometry device; S + B MedVET Babenhausen, Germany). Thoracic radiographs were digitally recorded in three dimensions at T0, and off-line analysis of the VHS was done by the same observer (Liselotte B. Christiansen) using free and open source code software (Horos Viewer for Mac OS X, Nimble Co LLC, MD, USA).

### 2.3. Study Medication

The Q10 formulation was gelatin capsules containing ubiquinone dissolved in vegetable oil at a dosage of 100 mg/dog twice daily. Identical placebo capsules were produced, and both products were manufactured according to Good Manufacturing Practices (GMP) guidelines and supplied in identical sealed, screw-top containers provided by PharmaNord Aps (Vejle, Denmark). Owners received 50 capsules, enough for three weeks, with four days’ supply to spare. Owners were instructed to note each treatment and the presence of any adverse reactions observed on a designated chart and to obtain their dog’s respiratory sleeping rate once every day. Adherence was assessed by counting the remaining capsules that were in the containers by the owners and were calculated using the formula: % Adherence = 100 × (number of dispensed capsules minus the number of returned capsules)/number of prescribed tablets.

### 2.4. Blood Sampling and Blood Analyses

A jugular venous blood sample was drawn into two serum separator clot activator tubes, one heparin tube, and two dipotassium ethylenediaminetetraacetic acid (K_2_EDTA) tubes using a 21 gauge butterfly catheter connected to a vacutainer system. Heparinized tubes were kept on wet ice in a lightproof container until centrifugation to prevent ex vivo oxidation of Q10 [22]. Within 30 min for the heparinized blood and one K_2_EDTA tube, and after 30 min for the serum tube, blood was centrifuged at 3000× *g* for 10 min at 4 °C and the supernatants were aliquoted into microcentrifuge tubes. One portion of serum and 1 K_2_EDTA tube of stabilized whole blood were delivered to the Veterinary Diagnostic Laboratory (Department of Veterinary Clinical Sciences, University of Copenhagen, Frederiksberg, Denmark) for evaluation of complete blood count (CBC) and a standard biochemistry profile. A manual platelet count was performed after adding 20 µL of ethylenediaminetetraacetic acid (EDTA)-stabilized blood to 380 µL of stromatolytic solution, as previously described [23]. The remaining serum and plasma aliquots were immediately stored at −80 °C for later batch analyses. Serum concentrations of the cardiac myocyte injury marker cardiac troponin-I (cTnI) were analyzed in duplicates using the sensitive Siemens ADVIA Centaur cTnI-ultra assay [24].

EDTA stabilized plasma was shipped to a reference laboratory on dry ice, and concentrations of the cardiac biomarker N-terminal pro-brain natriuretic peptide (NT-proBNP) were analyzed in duplicates using a commercial ELISA (Idexx Bioresearch, Ludwigsburg, Germany). 

### 2.5. Echocardiography

Standardized transthoracic echocardiographic views [25] were recorded from the right parasternal and left apical windows by one trained examiner (Lisbeth H. Olsen) using a Vivid E95 echocardiographic system with a 6S transducer (GE Healthcare Denmark, Brøndby, Denmark) and with continuous electrocardiography (ECG) recording. A standardized echocardiographic protocol using 2-D, M-mode, Color Doppler, and spectral Doppler was followed, as previously described [26].

At the first visit, the bodyweight normalized left ventricular internal diameter in diastole (LVIDDN) and left atrial-to-aortic ratio (LA/Ao) were determined for the purpose of staging the dog into ACVIM class. This was done on five consecutive cardiac cycles off-line after the examination. Left atrial (LA) and aortic (Ao) diameters were measured at the right parasternal short-axis view, and a ratio calculated [27]. The internal diameter of the left ventricle in diastole (LVIDD) was measured and normalized to bodyweight using 2D guided M-mode at the right parasternal short-axis view [28]. 

Fractional shortening was calculated from left ventricular dimensions obtained using M-mode [29] and the ejection fraction was calculated from measurements obtained using a 2D right parasternal long-axis view, as previously described [30]. All echocardiographic measurements were done off-line using EchoPac software (EchoPAC PC. Version 202, GE Healthcare Denmark, Brøndby, Denmark) with the observer (Maria Josefine Reimann) blinded to the treatment (Q10 or placebo).

### 2.6. Pharmacokinetic Assessment

In six of the dogs, blood samples were drawn from the jugular vein into one heparinized tube on five consecutive days following a treatment period. The first blood sample was taken in the morning following the last administration of Q10, and the following blood samples were obtained with 24 h interval. All the participating dog owners were invited to deliver the additional blood samples from their dogs, and six of them came for the extra visits with their dog. The blood samples were scheduled by the veterinarian (LBC) to take place following the treatment phase with Q10 without notification of the owner of the treatment sequence. 

### 2.7. Q10 Concentration by High-Performance Liquid Chromatography-Electrochemical Detection

Heparinized plasma samples obtained at the end of each treatment period in all dogs and from six dogs on five consecutive days following Q10 treatment were analyzed for Q10 concentrations (total, reduced, and oxidized Q10) using high-performance liquid chromatography (HPLC) with electrochemical detection, as previously described [17]. 

In brief, 75 µL of 1-propanol and 10 µL BHT (10 mg/mL in 96% ethanol) was added to 25 µL of canine plasma. The solution was mixed and centrifugation at 16,000× *g* in 2 min at 4 °C. The supernatant was analyzed immediately in duplicate on an HPLC system equipped with a Waters Nova-pak C_18_ column (150 × 3.9 mm; 4 µm, 60 Å) employing a mobile phase consisting of 20 mM LiPerChl·3H_2_O dissolved in ethanol/methanol/2-propanol (75:16.7:8.3, *v/v/v*). Detection was achieved using an RS6011 ultra-analytical cell (Thermo Scientific, Waltham, MA, USA) set at 500 mV as specified in detail elsewhere [17]. Intra- and inter-day precisions were below 6.5% and recoveries were between 89 and 109%. 

### 2.8. Statistical Analyses

Statistical analyses were conducted using statistical software (SAS version 9.4, SAS Institute Inc., Cary, NC, USA) with a significance level set at *p* < 0.05. Graphics of data were designed using GraphPad Prism (Version 8.0, La Jolla, CA, USA) or Microsoft Excel (version 15.37, Microsoft Corporation, Redmond, WA, USA) for pharmacokinetic data. Data are presented as medians and interquartile ranges (IQR) unless otherwise stated.

Basic characteristics of the dogs randomized to either treatment sequence were compared using non-parametric tests: Continuous variables were tested using the Wilcoxon signed-rank sum test, and categorical data were compared using Fisher’s exact test. 

A linear mixed model with backward reduction, taking repeated measurements into account, was applied to test if there was an effect of Q10 treatment and treatment sequence (Q10 or placebo first) on the primary and secondary endpoints. These were Q10 concentrations in plasma (total, oxidized, and reduced, respectively), the oxidation rate of Q10 (percentage of total Q10 that was oxidized), echocardiographic markers and circulating biomarkers of disease severity, serum biochemistry, and QoL score. Treatment (Q10 or placebo), treatment sequence, and the interaction between them were class variables, and in addition, the baseline value (T0) was included as explanatory variables. The dog was included as a random variable in the statistical model. 

A linear model was applied with the purpose of testing if there was a disease progression or a carry-over effect from the first baseline measurement (T0) to the second baseline measurement (T2). This was done by testing if the delta value between T2 and T0 was significantly different from zero. The same model was used to test if a difference between T0 and T2 was dependent on whether Q10 or placebo was administered in the first sequence.

The residuals of the linear models were tested for normality using a Shapiro–Wilks test in combination with assessment of homogeneity of variances by visual inspection of residuals, QQ plots, and histograms. Where necessary, transformations of response variables using either the natural logarithm, square root, or the inverse value were done to obtain the best fit for the linear model.

## 3. Results

### 3.1. Study Population

From November 2017 to November 2018, 34 CKCS were screened for eligibility to participate in the study. Nineteen dogs met the inclusion criteria and were randomized to receive either Q10 (*n* = 9) or placebo (*n* = 10) in the first phase of the study. One of the dogs (ACVIM group C) was subsequently excluded, due to early treatment discontinuation during the first treatment phase (placebo) because of undefined clinical signs of discomfort reported by the dog owner. No data from this dog were included in the analyses. The basic characteristics of the remaining 18 dogs are shown in Table 1. One dog (ACVIM group B2) was withdrawn from the study during the wash out period following the first treatment phase (placebo). This dog turned ill acutely and was euthanized in primary practice after being diagnosed with giardiasis. Data obtained at T0 and T1 for this dog were included in the analysis with data points from T2 and T3 as missing data. Seventeen dogs completed the entire study. 

The dogs randomized to each of the two treatment sequences were similar with respect to age, sex distribution, ACVIM class, use of cardiac medication, BW, echocardiographic parameters, hematology and serum biochemistry parameters, total Q10 plasma concentration, and oxidation rate of Q10 (Table 1). The dogs randomized to receive Q10 in the first phase had significantly higher concentrations of cTnI (*p* = 0.03) and NT-proBNP (*p* = 0.03) at baseline (T0) compared to dogs receiving placebo in the first phase of the study and had a significantly higher QoL score (indicating a decrease in QoL).

Out of the 18 dogs included in the analyses, 10 were classified as ACVIM stage B2, and eight dogs were ACVIM stage C. One dog in stage B2 was treated with pimobendan. Other B2 dogs did not receive medication. Stage C dogs were all receiving diuretic treatment (furosemide), and pimobendan, and two dogs were additionally receiving an angiotensin-converting enzyme-inhibitor (benazepril) and spironolactone. Three of the participating dogs were treated with non-steroidal anti-inflammatory drugs (meloxicam (*n* = 2) and robenacoxib (*n* = 1)) for control of osteoarthritis. One dog was treated with pregabalin for the management of neuropathic pain.

### 3.2. Adherence

There were no reports of adverse reactions, and all the owners reported that the capsules were easy to administrate to their dogs. Based on counts of returned capsules, the median adherence was 100% (IQR 99.4–102.1) in the first phase of the study and 100% (IQR 97.5–100) in the second phase of the study.

### 3.3. Plasma Q10 Concentrations 

The total Q10 concentration in plasma at baseline (T0) was 0.92 (0.70–1.26) µg/mL.

**Figure 2:**
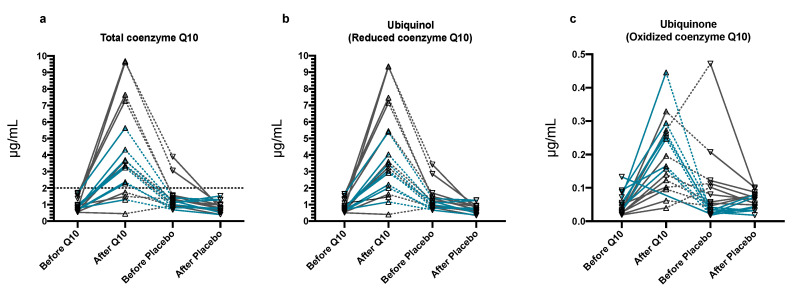
Plasma concentrations of (**a**) total Q10, (**b**) ubiquinone, and (**c**) ubiquinol before and after treatment with Q10 and placebo. Black lines denote dogs treated with Q10 first, then placebo. Blue lines denote dogs treated with a placebo first, then Q10. Dotted lines indicate the washout period. The vertical line indicates a cut-off of 2.0 μg/mL total Q10 in plasma that was the primary endpoint in the study. Before Q10: *n* = 17, after Q10: *n* = 16, before placebo: *n* = 18, after placebo: *n* = 16.

Plasma concentrations of total Q10 increased significantly (*p* < 0.0001) after three weeks of Q10 treatment compared to placebo (Figure 2a). The maximum concentration (Cmax) of total Q10 following supplementation was 3.51 (2.30–6.88) µg/mL. Thirteen dogs reached the primary endpoint of a total Q10 plasma concentration of ≥2.0 µg/mL (Figure 2a).

The concentrations of reduced Q10 in plasma increased significantly with Q10 treatment (*p* < 0.0001, Figure 2b) compared to placebo, as did the concentration of oxidized Q10 in plasma (*p* < 0.0001, Figure 2c). At baseline (T0), the Q10 oxidation rate was 3.2 (2.2–3.6)%. Treatment with Q10 or placebo did not cause the oxidation rate of Q10 to change, but the statistical analyses showed an increased oxidation rate of Q10 in the second treatment phase compared to the first treatment phase (*p* = 0.03) independent of which treatment was used (Q10 or placebo). This change in oxidation rate over time was driven by a statistically significantly higher concentration of oxidized Q10 in the second treatment phase (*p* < 0.0001) compared to the first phase that was occurring for unknown reasons.

Comparison of plasma concentrations of Q10 between the two baseline examinations (T0 and T2) revealed that dogs treated with Q10 in the first treatment phase had a significantly higher concentration of total and reduced Q10 in plasma at T2 compared to T0 (both *p* = 0.009) indicating a carry-over effect. The Q10 oxidation rate was not significantly different between T0 and T2 (*p* = 0.055). 

In the two dogs reaching the highest concentrations of total Q10 (9.6 and 9.7 µg/mL) after treatment with Q10, the total circulating Q10 concentration remained elevated at T2, indicating that the washout period was not sufficient to reach baseline concentrations. On the last examination (T3), both dogs had returned to a plasma concentration below their baseline concentrations (T0). 

### 3.4. Pharmacokinetic Analyses

**Figure 3:**
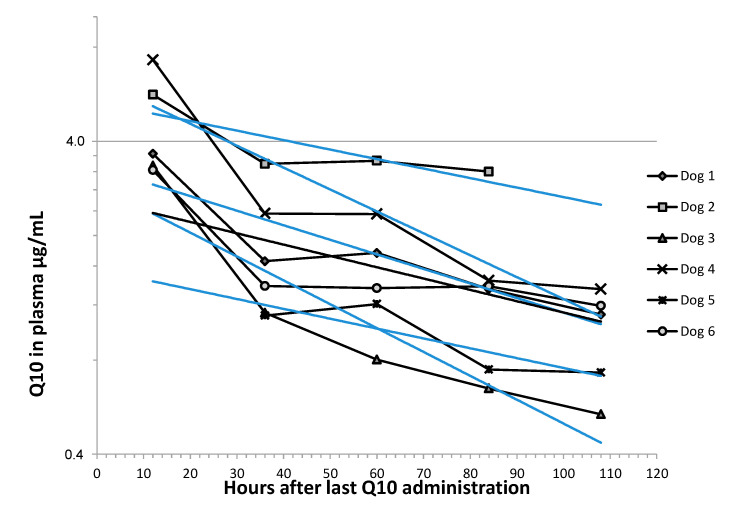
Elimination curves of plasma total Q10 against time. The plot shows plasma concentration of total Q10 in six dogs after 12, 36, 48, 60, and 72 h after the last treatment with Q10 (time 0) on a semilogarithmic scale.

Figure 3 shows concentration vs. time observations for six dogs after discontinuation of Q10 administration. The elimination phase of Q10 in plasma was assumed to follow first order kinetics, as previously described in dogs [31]. The elimination rate constant was estimated for each dog using exponential regression and used to calculate the half-life (T_1/2_) of Q10 from the formula T_1/2_ = ln(2)/k. An average T_1/2_ of 2.95 (IQR 1.75–4.02) days was found based on the elimination curves in six dogs. Two data points were missing in the analyses, due to sampling of heparinized blood being unsuccessful in one dog and an analytical error in one plasma sample (Figure 3).

### 3.5. Secondary Endpoints 

Treatment with Q10 did not significantly change echocardiographic parameters compared to placebo. Bodyweight, QoL score, serum concentrations of cholesterol, alanine aminotransferase (ALT) and creatinine, circulating biomarkers cTnI and NT-proBNP, hematocrit, and platelet count were all unchanged by treatment with Q10 compared to placebo (Table S1). In two dogs, data were missing for NT-proBNP, due to unsuccessful EDTA blood sampling. In one dog, no blood could be drawn at its second visit, resulting in missing data on all blood parameters from this dog. Five data points were missing in plasma concentrations of Q10: Sampling of heparinized blood was unsuccessful after the placebo treatment period in two dogs, one dog that was discontinued during the washout period missed data from before and after placebo treatment, and in one dog, there was an analytical error causing the plasma concentration after Q10 treatment to be missing (Figure 1 and Figure 2).

Comparison of echocardiographic parameters, circulating biomarkers, and QoL score at examinations T0 and T2 revealed no statistically significant changes, indicating no disease progression between the time points.

## 4. Discussion

Several formulations of Q10 are on the market, but dose recommendations to dogs are based on an anecdotal background. In this placebo-controlled clinical trial, we investigated plasma concentrations of Q10 in CKCS with severe MMVD before and after three weeks supplementation with Q10. The solubilized formulation of Q10 was well tolerated in all dogs. The primary endpoint of the total Q10 plasma concentration of ≥2.0 μg/mL was reached in 13 out of 16 dogs. A 3-week period of treatment with Q10 did not significantly improve echocardiographic indices of MMVD severity, circulating cardiac biomarkers, or owner perceived QoL compared to placebo. Pharmacokinetic analyses resulted in an estimated terminal T_1/2_ of 2.95 days. Our results are relevant when considering treatment dose and dose interval of Q10 in dogs and for the planning of future clinical trials of longer duration.

In 1998, Langsjoen and Langsjoen defined the therapeutic target concentration of Q10 to human patients with heart failure to be at least 2.0 μg/mL, which is above the endogenous circulating concentration [14]. Although others have suggested a higher therapeutic concentration for effective treatment of patients with heart failure (2.4 μg/mL in a human clinical trial) [32], the 2.0 μg/mL cut-off has been widely accepted. In experimental dogs, Harker-Murray et al. showed a positive effect of Q10 treatment in pacing-induced heart failure when reaching a circulating Q10 concentration of 2.0 μg/mL [33]. In the aforementioned Q-SYMBIO study, showing a positive effect on major cardiovascular outcomes in patients treated with Q10 compared to placebo [18], the 2.0 μg/mL target concentration of total Q10 was also applied. 

Endogenous concentrations of circulating Q10 in laboratory-bred [31,33,34,35] and privately-owned dogs [16,17] were similar to human endogenous reference levels [15]. On this background, we applied the 2.0 μg/mL concentration as the primary endpoint. All dogs had endogenous concentrations below this concentration before treatment. This is in line with previous studies and suggests a concentration of Q10 above the endogenous level being necessary for the distribution of Q10 to tissues [14]. 

Three dogs did not fulfill the primary endpoint. For two reasons, this was unexpected. First, the dose (100 mg twice daily per dog, corresponding to a mean dose of 23 mg/kg/day) of Q10 relative to BW was comparably higher than the 300 mg/day (corresponding to an average daily dose of 4 mg/kg/day) used in adult humans in a recent clinical trial [18]. When designing the study, the relatively high dose was chosen to ensure a considerable increase in plasma Q10. It has been shown previously that up to a certain level, the circulating Q10 increases with increasing daily oral dose [5]. In dogs, a no adverse effect level of Q10 of 6000 mg/kg/day has previously been determined [31], indicating that Q10 is safe. However, when designing the trial, choosing the same dose to all the dogs was unattended our finding of a considerable individual variation in plasma concentrations of Q10 following treatment. Secondly, we included only purebred CKCS in order to acquire a homogenous population. The baseline endogenous concentrations of Q10 among the CKCS were within a narrow range and comparable to what has been measured in healthy, adult CKCS under identical analytical conditions [17]. Two dogs in the study reached plasma concentrations >9 μg/mL of total Q10, but no obvious similarities in baseline characteristics for these two dogs can explain this variation in plasma concentrations of Q10. Both dogs were males, classified as ACVIM stages C and B2, respectively, and one of them received other cardiac medication. 

A high inter-individual variation in the circulating concentrations of total Q10 after treatment with Q10 was previously shown both in single-dose studies in healthy human volunteers [36,37], and in patients with congestive heart failure [32,38,39]. Our similar findings in dogs with MMVD suggest that the same applies to dogs. Because adherence in the study was very good, and owners found it easy to administrate the capsules to dogs, the limited plasma concentration of Q10 in some of the dogs may indicate saturation of intestinal absorption. The hydrophobic properties of Q10 are limiting for intestinal absorption, but the feeding of a fatty meal can enhance absorption [40]. We advised owners to deliver Q10 to their dogs with a meal, but the feeding regime was not standardized, and this may, at least in part, underlie the variability in plasma Q10 concentrations following treatment among the dogs. At this point, it is largely unknown which physiological factors determine the bioavailability of Q10 in individual human beings and in dogs. Differences in gut microbiota among subjects, edema in the gastrointestinal tract of heart failure patients, or differences in enterocyte metabolism are factors that were previously discussed to underlie the unequal bioavailability of Q10 in human subjects [14,36], and the same may be true in dogs, but needs further investigation.

The in vivo oxidation rate of Q10 (i.e., the percentage of total Q10 that is oxidized) is considered a relevant marker of oxidative stress, but the careful treatment of blood samples has to be ensured to prevent ex vivo oxidation of blood samples [41]. We did not observe a change in oxidation rate after Q10 treatment compared to placebo, but intriguingly, a 2.5% higher oxidation rate of Q10 was found in the second treatment phase compared to the first phase. In each phase, half of the dogs were treated with Q10 and the other half with a placebo. Human data report that, on average, 96% of plasma Q10 is reduced, corresponding to an oxidation ratio of 4% [8], which is similar to the 3% measured in the dogs at baseline in the present study. The difference in oxidation rate cannot readily be explained. It is unlikely to be caused by disease progression, as there were no changes in echocardiographic parameters and biomarkers between the first and second phase of the study. Sampling and storage procedures were standardized, and the Q10 analyses were done in one batch with the analyst blinded to treatment and treatment phase. A previous study reported an oxidation rate of circulating Q10 in healthy CKCS of 1.9% [17] that were of a younger age than the CKCS with MMVD used in the present study. Although speculative at this point, aging may have contributed to the small numerical increase to 3% in the oxidation ratio, as this has been shown to occur in human subjects [8]. The presence of MMVD may also underlie the increased oxidation ratio of circulating Q10 in the dogs related to the presence of oxidative stress. The literature contains divergent results on the presence of oxidative stress in the circulation of dogs with naturally occurring valvular heart disease [42,43]. The use of Q10 as an antioxidant to neutralize reactive oxygen species in the circulation and in heart muscle, are highly relevant for further investigation.

In the veterinary literature, there are empirical recommendations for using Q10 as a supplement, especially in dogs with dilated cardiomyopathy [44], but there are no studies justifying the dose recommendations or showing effects on clinical parameters. Significant reductions in circulating concentrations of NT-proBNP and improvement of QoL were seen in the human Q-SYMBIO trial, using the same formulation of Q10 as used in the present study [18]. No similar significant improvements in NT-proBNP levels and QoL were observed in the dogs treated with Q10. Our study may have been underpowered or the treatment phases too short for a significant difference to occur in both treatment phases. A major difference between cardiovascular outcome trials done in human patients with a positive effect of Q10 and the present study in dogs is the duration of treatment. In the Q-SYMBIO trial, patients had been treated for two-years at the time a significant difference in cardiovascular outcome was observed, while at week 16 and 106, no significant improvements were observed. The CKCS in the present study were treated for only three weeks. In another human study, in which the pharmacokinetic parameters in the human subjects resembled our findings in dogs, there were also no statistically significant effects of Q10 on echocardiographic parameters after six months of treatment [38]. Although short-term daily treatment (12 weeks or less) with 100 mg CoQ10 has been shown to improve left ventricular ejection fraction and clinical signs of heart failure in studies in human patients [45], there seems to be agreement among human studies that a positive effect of Q10 on the heart requires chronic treatment and a minimum treatment period of three months has been suggested [46]. It is currently unknown if prolonged treatment with Q10 enhances cardiac health in dogs, while no controlled prospective studies have been undertaken.

Our study has some limitations. As mentioned previously, dog diets were not standardized, except for food fasting prior to obtaining blood samples. Additionally, some of the dogs received cardiac medication and other pharmaceuticals that cannot be excluded to affect pharmacokinetic parameters of Q10 in dogs, although their interactions with Q10 have not previously been described. Q10 is endogenously synthesized in cells but also supplied with food, with meat containing the highest amount [47]. However, it has been proposed that the endogenous, circulating concentration of Q10 is only very little affected by diet [48]. The baseline endogenous concentrations of Q10 among the dogs in the study were within a quite narrow range, and similar to concentrations reported previously in CKCS and other breeds [16,17]. We, therefore, gather it unlikely that the diets had a significant impact on endogenous Q10 concentrations. The composition of diets may, as previously described, have affected the absorption of Q10, which in turn could cause the variation in Q10 plasma concentrations seen among the CKCS. This factor may be limiting the possibility of generalizing the data to other dog populations.

This study was pioneering for determining the absorption and pharmacokinetics of a commercially available formulation of Q10 in dogs and for investigating a possible efficacy on clinical parameters related to MMVD. The number of included dogs was, therefore, not determined from power calculations. We did observe a significant increase in plasma concentrations after oral delivery with Q10, indicating that for this purpose, the population was large enough. For observing improvements in clinical parameters of MMVD, the study may have been underpowered and likely of too short duration. Our study provides data that can be included in the calculation of sample sizes in future prospective studies.

## 5. Conclusions

In conclusion, we show that a gelatin capsule formulation of solubilized Q10 is well tolerated in CKCS with MMVD and is absorbed into the circulation without adverse reactions. There were, however, inter-individual variations in the plasma concentrations with this formulation of Q10 similarly to findings in humans. A dosage of 200 mg/day for three weeks of Q10 does not appear to change the clinical severity of MMVD or QoL in CKCS. The estimated T_1/2_ of 2.95 days may suggest that every other day treatment in dogs may suffice to keep circulating concentrations of Q10 ≥ 2.0 μg/mL in dogs.

The variation in Q10 plasma concentrations observed among the CKCS suggests initiation of studies investigating the physiological factors determining intestinal absorption of Q10, including dosing and feeding regimens.

A recent study showed that the use of antioxidants (Q10 not included) and fatty acids slowed the progression of spontaneous MMVD in dogs [49]. Ultimately, investigation of long-term use of Q10 in dogs with MMVD and heart diseases of other etiologies are therefore relevant—preferably with dosing tailored in accordance with laboratory testing of Q10 plasma concentrations.

## Figures and Tables

**Table 1:** Baseline demographics and clinical characteristics of the 18 Cavalier King Charles Spaniels included in the statistical analyses. Dogs were either randomized to receive Q10 first, then placebo (Q10 in phase 1) or to receive placebo first, then Q10 (Q10 in phase 2).

Group	Dogs Randomized to Receive Q10 in Phase 1	Dogs Randomized to Receive Q10 in Phase 2	*p*-Value
	*N* = 9	*N* = 9	
Sex male/female	6/3	7/2	>0.999
Murmur intensity 3/4/5	0/6/3	1/7/1	0.58
Age (years)	9.2 (9.1–10.0)	8.7 (8.3–9.0)	0.16
Bodyweight (kg)	8.6 (8.4–9.3)	9.0 (8.0–9.6)	0.69
ACVIM (B2/C)	5/4	5/4	1.0
Quality of Life (QoL) score	9 (9–11)	12 (12–14)	0.015 *
Heart rate (bpm)	124 (120–140)	112 (100–128)	0.17
Systolic blood pressure (mmHg)	145 (126–151)	147 (143–167)	0.29
VHS	12.3 (11.6–12.4)	11.8 (11.5–12)	0.14
LVIDDN	2.0 (1.9–2.5)	1.9 (1.8–2.0)	0.20
FS (%)	40.6 (40–43.3)	40.9 (36.3–46.1)	0.70
EF (%)	78.8 (76.4–80.2)	77.5 (74.3–80.5)	0.96
LA/Ao	2.1 (1.8–2.5)	1.8 (1.6–2.0)	0.10
Hematocrit	40.2 (38.4–41.2)	43.7 (38.5–44.5)	0.18
Platelet count (× 10^6^ platelets/mL)	311 (245–372)	253 (105–272)	0.35
Creatinine (μmol/L)	63 (58–67)	60 (54–78)	0.79
ALT (U/L)	43 (35–52)	51 (38–60)	0.66
NT-proBNP, ng/mL	1851 (1577–3438)	1158 (998–1282)	0.029 *
cTnI (ng/mL)	0.052 (0.039–0.074)	0.028 (0.027–0.032)	0.027 *
Total Q10 concentration in plasma (μg/mL)	0.85 (0.63–1.36)	1.00 (0.82–1.14)	0.66
Oxidation ratio (%)	3.38 (2.29–3.58)	3.07 (2.45–3.25)	0.60

Data are presented as median (interquartile range). * denotes statistically significant differences between groups. *p*-values were calculated using a Wilcoxon signed-rank sum test and Fisher’s exact test for categorical data. Abbreviations: ACVIM, American College of Veterinary Internal Medicine; FS, Fractional Shortening; EF, Ejection Fraction; LVIDDN, Left ventricular internal diameter in diastole normalized for bodyweight; LA/Ao, Left atrial to aortic root ratio; ALT, alanine aminotransferase; NT-proBNP; N terminal pro-B type natriuretic peptide; cTnI; cardiac troponin I; VHS, vertebral heart score.

## Supplementary Materials

The following are available online at https://www.mdpi.com/2076-3921/9/9/827/s1, Table S1. Data for secondary clinical endpoint at each of the visits following baseline visit (T0). Data are shown for each of the group: dogs randomized to receive Q10 first, then placebo (Q10 first) and dogs randomized to receive placebo first, then Q10 (Placebo first).

## References

[1] Egenvall A., Bonnett B.N., Häggström J. (2006). Heart disease as a cause of death in insured Swedish dogs younger than 10 years of age. J. Vet. Intern. Med..

[2] Darke P. (1987). Valvular incompetence in cavalier King Charles spaniels. Vet. Rec..

[3] Häggström J., Hansson K., Kvart C., Swenson L. (1992). Chronic valvular disease in the cavalier King Charles spaniel in Sweden. Vet. Rec..

[4] Häggström J., Boswood A., O’Grady M., Jons O., Smith S., Swift S., Borgarelli M., Gavaghan B., Kresken J.-G., Patteson M.W. (2008). Effect of Pimobendan or Benazepril Hydrochloride on Survival Times in Dogs with Congestive Heart Failure Caused by Naturally Occurring Myxomatous Mitral Valve Disease: The QUEST Study. J. Vet. Intern. Med..

[5] Bhagavan H.N., Chopra R.K. (2007). Plasma coenzyme Q10 response to oral ingestion of coenzyme Q10 formulations. Mitochondrion.

[6] Lenaz G., Fato R., Formiggini G., Genova M.L. (2007). The role of Coenzyme Q in mitochondrial electron transport. Mitochondrion.

[7] Bentinger M., Brismar K., Dallner G. (2007). The antioxidant role of coenzyme Q. Mitochondrion.

[8] Miles M.V., Horn P.S., Morrison J.A., Tang P.H., Degrauw T., Pesce A.J. (2003). Plasma coenzyme Q10 reference intervals, but not redox status, are affected by gender and race in self-reported healthy adults. Clin. Chim. Acta.

[9] Stride N., Larsen S., Hey-Mogensen M., Sander K., Lund J.T., Gustafsson F., Køber L., Dela F. (2013). Decreased mitochondrial oxidative phosphorylation capacity in the human heart with left ventricular systolic dysfunction. Eur. J. Heart Fail..

[10] Sharov V.G., Goussev A., Lesch M., Goldstein S., Sabbah H.N. (1998). Abnormal Mitochondrial Function in Myocardium of Dogs with Chronic Heart Failure. J. Mol. Cell. Cardiol..

[11] Neubauer S. (2007). The Failing Heart—An Engine Out of Fuel. N. Engl. J. Med..

[12] Folkers K., Vadhanavikit S., Mortensen S.A. (1985). Biochemical rationale and myocardial tissue data on the effective therapy of cardiomyopathy with coenzyme Q10. Proc. Natl. Acad. Sci. USA.

[13] Mantle D., Dybring A. (2020). Bioavailability of Coenzyme Q10: An Overview of the Absorption Process and Subsequent Metabolism. Antioxidants.

[14] Langsjoen P.H., Langsjoen A.M. (1998). Coenzyme Q10 in cardiovascular disease with emphasis on heart failure and myocardial ischaemia. Asia Pac. Heart J..

[15] Sharma A., Fonarow G.C., Butler J., Ezekowitz J.A., Felker G.M. (2016). Coenzyme Q10 and heart failure—A state-of theart Review. Circ. Heart Fail..

[16] Svete A.N., Verk B., Seliškar A., Tomsič K., Križman P.J., Petrič A.D. (2017). Plasma coenzyme Q10concentration, antioxidant status, and serum N-terminal pro-brain natriuretic peptide concentration in dogs with various cardiovascular diseases and the effect of cardiac treatment on measured variables. Am. J. Vet. Res..

[17] Schou-Pedersen A.M.V., Schemeth D., Lykkesfeldt J. (2019). Determination of Reduced and Oxidized Coenzyme Q10 in Canine Plasma and Heart Tissue by HPLC-ECD: Comparison with LC-MS/MS Quantification. Antioxidants.

[18] Mortensen S.A., Rosenfeldt F., Kumar A., Dolliner P., Filipiak K.J., Pella D., Alehagen U., Steurer G., Littarru G.P., Q-SYMBIO Study Investigators (2014). The Effect of Coenzyme Q 10 on Morbidity and Mortality in Chronic Heart Failure. JACC Heart Fail..

[19] Boswood A., Häggström J., Gordon S., Wess G., Stepien R., Oyama M., Keene B., Bonagura J., Macdonald K., Patteson M.W. (2016). Effect of Pimobendan in Dogs with Preclinical Myxomatous Mitral Valve Disease and Cardiomegaly: The EPIC Study-A Randomized Clinical Trial. J. Vet. Intern. Med..

[20] http://www.randomization.com.

[21] Schulz K.F., Altman D.G., Moher D., Consort Group (2010). CONSORT 2010 Statement: Updated Guidelines for Reporting Parallel Group Randomised Trials. BMJ.

[22] Tang P.H., Miles M.V., Steele P., Degrauw A., Chuck G., Schroer L., Pesce A. (2002). Anticoagulant effects on plasma coenzyme Q(10) estimated by HPLC with coulometric detection. Clin. Chim. Acta.

[23] Eksell P., Häggström J., Kvart C., Karlsson A. (1994). Thrombocytopenia in the cavalier King Charles spaniel. J. Small Anim. Pr..

[24] Langhorn R., Willesen J.L., Tarnow I., Kjelgaard-Hansen M. (2013). Evaluation of a high-sensitivity assay for measurement of canine and feline serum cardiac troponin I. Vet. Clin. Pathol..

[25] Thomas W.P., Gaber C.E., Jacobs G.J., Kaplan P.M., Lombard C.W., Vet M., Moise N.S., Moses B.L. (1993). Recommendations for Standards in Transthoracic Two-Dimensional Echocardiography in the Dog and Cat. J. Vet. Intern. Med..

[26] Reimann M., Moller J.E., Häggström J., Markussen B., Holen A., Falk T., Olsen L. (2014). R-R interval variations influence the degree of mitral regurgitation in dogs with myxomatous mitral valve disease. Vet. J..

[27] Häggström J., Hansson K., Karlberg B.E., Kvart C., Olsson K. (1994). Plasma concentration of atrial natriuretic peptide in relation to severity of mitral regurgitation in Cavalier King Charles Spaniels. Am. J. Vet. Res..

[28] Cornell C.C., Kittleson M.D., Della Torre P., Häggström J., Lombard C.W., Pedersen H.D., Vollmar A., Wey A. (2004). Allometric scaling of M-mode cardiac measurements in normal adult dogs. J. Vet. Intern. Med..

[29] Lombard C.W. (1984). Normal values of the canine M-mode echocardiogram. Am. J. Vet. Res..

[30] Wess G., Maurer J., Simak J., Hartmann K. (2010). Use of Simpson’s Method of Disc to Detect Early Echocardiographic Changes in Doberman Pinschers with Dilated Cardiomyopathy. J. Vet. Intern. Med..

[31] Yerramilli-Rao P., Beal M.F., Watanabe D., Kieburtz K., De Blieck E.A., Kitano M., Hosoe K., Funahashi I., Cudkowicz M.E. (2012). Oral Repeated-Dose Toxicity Studies of Coenzyme Q10 in Beagle Dogs. Int. J. Toxicol..

[32] Belardinelli R., Lacalaprice F., Solenghi M., Seddaiu G., Principi F., Tiano L., Littarru G.P., Muçaj A. (2006). Coenzyme Q10 and exercise training in chronic heart failure. Eur. Heart J..

[33] Harker-Murray A.K., Tajik A., Ishikura F., Meyer D., Burnett J.C., Redfield M.M. (2000). The role of coenzyme Q10 in the pathophysiology and therapy of experimental congestive heart failure in the dog. J. Card. Fail..

[34] Yuan B., Liu C., Xu P., Lin L., Pan C., Wang L., Xu H. (2010). Validated HPLC method for the quantitative determination of CoQ10 in dog plasma and its application to a pharmacokinetic study. Biomed. Chromatogr..

[35] Zaghloul A.-A., Gurley B., Khan M.A., Bhagavan H., Chopra R., Reddy I.K. (2002). Bioavailability Assessment of Oral Coenzyme Q10 Formulations in Dogs. Drug Dev. Ind. Pharm..

[36] Lopez-Lluch G., Del Pozo-Cruz J., Sánchez-Cuesta A., Cortés-Rodríguez A.B., Navas P. (2019). Bioavailability of coenzyme Q10 supplements depends on carrier lipids and solubilization. Nutrition.

[37] Vitetta L., Leong A., Zhou J., Forno S.D., Hall S., Rutolo D. (2018). The Plasma Bioavailability of Coenzyme Q10 Absorbed from the Gut and the Oral Mucosa. J. Funct. Biomater..

[38] Khatta M., Alexander B.S., Krichten C.M., Fisher M.L., Freudenberger R., Robinson S.W., Gottlieb S.S. (2000). The effect of coenzyme Q10 in patients with congestive heart failure. Ann. Intern. Med..

[39] Munkholm H., Hansen H.H.T., Rasmussen K. (1999). Coenzyme Q10 treatment in serious heart failure. BioFactors.

[40] Ochiai A., Itagaki S., Kurokawa T., Kobayashi M., Hirano T., Iseki K. (2007). Improvement in intestinal coenzyme q10 absorption by food intake. Yakugaku Zasshi.

[41] Lagendijk J., Ubbink B., Vermaak W.J.H. (1996). Measurement of the ratio between the reduced and oxidized forms of coenzyme Ql0 in human plasma as a possible marker of oxidative stress. J. Lipid Res..

[42] Freeman L.M., Rush J.E., Milbury P.E., Blumberg J.B. (2005). Antioxidant status and biomarkers of oxidative stress in dogs with congestive heart failure. J. Vet. Intern. Med..

[43] Reimann M., Häggström J., Moller J.E., Lykkesfeldt J., Falk T., Olsen L. (2017). Markers of Oxidative Stress in Dogs with Myxomatous Mitral Valve Disease are Influenced by Sex, Neuter Status, and Serum Cholesterol Concentration. J. Vet. Intern. Med..

[44] Freeman L.M. (1998). Interventional nutrition for cardiac disease. Clin. Tech. Small Anim. Pr..

[45] Fotino A.D., Thompson-Paul A.M., Bazzano L.A. (2012). Effect of coenzyme Q₁₀ supplementation on heart failure: A meta-analysis. Am. J. Clin. Nutr..

[46] Langsjoen P.H. (2000). Lack of effect of coenzyme Q on left ventricular function in patients with congestive heart failure. J. Am. Coll. Cardiol..

[47] Weber C., Bysted A., Hølmer G. (1997). Coenzyme Q10 in the diet-daily intake and relative bioavailability. Mol. Asp. Med..

[48] Miles M.V., Horn P., Miles L., Tang P., Steele P., Degrauw T. (2002). Bioequivalence of coenzyme Q10 from over-the-counter supplements. Nutr. Res..

[49] Li Q., Heaney A., Langenfeld-McCoy N., Boler B.V., Laflamme D.P. (2019). Dietary intervention reduces left atrial enlargement in dogs with early preclinical myxomatous mitral valve disease: A blinded randomized controlled study in 36 dogs. BMC Vet. Res..

