# Supplementary material for: Pharmacokinetics of Repeated Oral Dosing with Coenzyme Q10 in Cavalier King Charles Spaniels with Myxomatous Mitral Valve Disease
Source: Antioxidants (Basel). 2020 Sep 4;9(9):827. doi: 10.3390/antiox9090827 (PMC7555137; doi:10.3390/antiox9090827)
Supplement: Supplementary file 1 [file antioxidants-09-00827-s001.pdf]

Table 1. Data for secondary clinical endpoint at each of the visits following baseline visit (T0). Data are shown for each of the group: dogs randomized to receive Q10 first, then placebo (Q10 first) and dogs randomized to receive placebo first, then Q10 (Placebo first).

**Clinical Visit**	**T1**		**T2**		**T3**	
**Treatment Sequence**	**Q10 first**	**Placebo first**	**Q10 first**	**Placebo first**	**Q10 first**	**Placebo first**
QoL	11.0 (10.0–11.0)	9.0 (8.0–10.0)	12.0 (11.0–13.0)	9.5 (8.0–11.8)	13.0 (12.0–13.0)	8.5 (8.0–12.3)
LVIDDN	2.1 (1.8–2.2)	1.8 (1.7–1.9)	2.1 (1.7–2.1)	1.9 (1.8–1.9)	2.0 (1.8–2.1)	1.8 (1.7–1.9)
FS (%)	40.0 (34,8–41.3)	38.0 (36.7–42.4)	39.8 (36.9–44.4)	39.4 (34.4–43.3)	39.5 (33.7)	38.6 (35.5–44.8)
EF (%)	76.9 (74.3–78.6)	78.2 (64.5–78.8)	75.1 (70.3–78.0)	80.1 (72.9–80.8)	75.8 (70.8–78.8)	77.4 (74.1–81.2)
LA/Ao	2.0 (1.9–2.3)	1.7 (1.6–2.0)	2.2 (1.6–2.6)	1.9 (1.6–2.1)	2.1 (1.8–2.2)	1.7 (1.7–2.0)
Platelet count (x106 platelets /mL)	276.0 (174–395)	254 (200.5–350.5)	274 (190–412)	278 (196.9–330.3)	265 (198–365)	261.5 (191.8–310.8)
Creatinine (µmol/L)	61.0 (56.0–68.0)	67.5 (63.3–71)	58.0 (55.0–77.0)	68 (60–78.5)	61.0 (58.0–75.0)	63 (60.3–70.8)
ALT (U/L)	41.0 (35.0–59.0)	43.5 (39.3–54.0)	41.0 (38.0–59.0)	43.5 (36.5–72.5)	41.0 (36.0–54.0)	41.0 (35.8–45.6)
NT-proBNP, ng/mL	1717.5 (1408.5–2660.8)	1307.0 (1118.8–1421.5)	1830.0 (1611.0–3860.0)	1343 (830.3–1703.0)	2202.0 (1604.0–4154.0)	1168.5 (878.3–1431.3)
CTnI (ng/mL)	0.052(0.039–0.074)	0.025(0.021–0.027)	0.053(0.036–0.065)	0.022(0.020–0.027)	0.052(0.035–0.096)	0.024(0.019–0.034)

Data are presented as median (interquartile range). FS, Fractional Shortening; EF, Ejection Fraction; LVIDDN, Left ventricular internal diameter in diastole normalized for body weight; LA/Ao, Left atrial to aortic root ratio; ALT, alanine aminotransferase; NT-proBNP; N terminal pro-B type natriuretic peptide; cTnI; cardiac troponin I; VHS, vertebral heart score.
